# The unstudied effects of wording and answer formats in the analysis of impartiality in public service provision

**DOI:** 10.1371/journal.pone.0288977

**Published:** 2023-07-26

**Authors:** Juan Carlos Martín, Pedro Moreira, Concepción Román

**Affiliations:** Institute of Tourism and Sustainable Economic Development, Universidad de Las Palmas de Gran Canaria, Las Palmas de Gran Canaria, Spain; Universite Paris Pantheon-Assas, FRANCE

## Abstract

Impartiality in public services provision is an important dimension that explains the quality of government (QoG). The analysis of impartiality has boomed in recent years at different territorial levels, like countries or regions. The impartiality measures depend on several attributes that are aggregated using different methods. However, little attention has been given to the effects of negative wording attributes and the number of format answers, despite the efforts made by previous studies to build robust composite impartiality indices. This study corrects this existing gap partly using one of the most extensive surveys (the European Quality of Government Index 2021) that include attributes related to impartiality (six attributes and 129,991 citizens). The method will be based on a fuzzy clustering approach, the extended Apostle model and an ordinary binary probit model. The results show that the type of wording and the number of answer options affect impartiality. The analysis of the main differences observed is affected by some insightful covariates such as country, gender, being native, town size, occupation, and the perception of the economic situation.

## 1 Introduction

Impartiality has become a cornerstone in the multidisciplinary analysis of the quality of government (QoG), which is linked with the concept of corruption in political economy science [1,2]. Political corruption is usually defined as a breach of the norm of impartiality. There exists a number of potential power abuses that can be exerted at different public service offices that push into the citizens’ wrong governance perception. Citizens distrust the institutions when they observe that public services are administered with a lack of transparency that favours partiality and corruption [3,4].

Corruption and impartiality usually affect economic development, individual living standards, income distribution, economic resilience and subjective well-being, among other socioeconomic variables [5–8]. Thus, as good QoG seems to be connected with the national and individual performance, some international and national institutions have developed adequate surveys to analyse QoG. We can cite the most representative surveys: the World Governance Indicators, the Transparency International Corruption Index and the European Quality of Government Index.

Numerous studies have either used these datasets to study QoG or analyse how the QoG affects some socio-economic variables [4,9–12]. Charron et al. [1] contended that most of the QoG studies suffered from two key problems related to the underrepresented focus on analysing QoG at the subnational level and the overrepresented experts’ opinions in the datasets who provide direct information on the levels of corruption.

However, a third weakness that has been neglected so far is the negative wording of questions and the answer formats of these studies that could compromise the results obtained for some of the dimensions. In other fields, e.g. education, empirical evidence shows that response quality can be affected by cognitive abilities, acquiescence bias, and question difficulty [13]. This issue is relevant in the case under study as QoG indices have been extensively used by researchers and the European Commission to establish policy scenarios and strategies that enhance governance performance. Recent research shows that bad governance harms domestic redistributive efforts and citizens’ willingness to exchange tax money between member states [14].

Thus, the research questions of this study can be summarized as follows: (1) Are impartiality indices robust to negative wording questions and different answer formats? (2) Is it possible to determine whether some citizens provide more unreliable information than others? (3) Is there evidence that some of the potential inconsistency can be explained by some socio-demographic covariates of the respondents? Thus, the responses to the research questions contribute to shedding some light on an area underresearched such as the study of impartiality as an important component of the analysis of QoG.

The remainder of the paper is organized as follows: Section 2 offers some insights from the literature, section 3 describes the data section, section 4 details the methodology, section 5 presents and discusses the results, and section 6 offers some concluding remarks.

## 2 Literature review

Rothstein and Teorell [15] reviewed the existing research on “good governance” and the quality of government institutions. They believed that a clearer and more specific definition of the quality of government was needed and proposed that it be based on the impartiality of government institutions. Their main argument was that a democratic system has two opposing norms: partisanship for the representational process and impartiality for the implementation process. Aucoin [16] discussed for the first time the New Political Governance (NPG) as a transformation of the public service tradition of impartiality in the administration of public services and the nonpartisan management of the public services into a politicisation system that converted top public service posts and public servants into true political partisans. In a recent study by Suzuki and Demircioglu [17], it was discovered that countries with professional and impartial public administration tend to have higher levels of national innovation outputs. The research highlighted the significance of having such a system in place for boosting innovation.

Charron et al. [18] emphasised that impartiality is a crucial QoG component that explains several variables associated with the well-being of a country’s people, focusing more on the importance of "how" a government implements its policies in an impartial and corruption-free manner, rather than on "what" it delivers. The authors concluded that QoG varies significantly in four main groups of European states. The best performers are Scandinavian, Germanic and English-speaking countries, followed by the group of primarily Mediterranean countries together with Estonia and Slovenia. The third group consists of most of the new member states plus Italy and Greece. Finally, the group of the two newest EU member states, Romania and Bulgaria.

Considering the national dimension of 27 European Union (EU) countries and 172 subnational regions in 2008, the results showed significant variations within the same country, in federal or semi-federal nations such as Italy, Belgium or Spain, and more centralised countries, such as Portugal, Romania or Bulgaria. Other countries, such as Denmark, Poland, Austria and Slovakia, showed minimal variation. Another interesting finding was the positive relationship between the HDI (Human Development Index), the social trust and the QoG indicator, both within and between EU countries.

These results were already found in the literature where countries with high QoG score higher in almost all dimensions related to the well-being of their citizens [19]. They achieve better economic performance [20–22], greater environmental sustainability [23,24], lower income inequality and poverty [25], better education and health outcomes [26], higher levels of subjective happiness [27] and lower likelihood of armed civil conflict [28].

More recently, Suzuki & Hur [29] assimilated the QoG with that of meritocracy using a dataset of citizens living in 21 European countries. The authors analysed to what extent the citizens believe that success in public and private organisations is due to hard work rather than luck or social connections. They found that sectoral differences exist in almost all European regions, although these are smaller in countries where New Public Management (NPM) practices and meritocratic principles are established. Interestingly, in most regions and countries, perceived meritocracy in private organisations is higher than in public organisations. They also observed that in countries where NPM and meritocracy are well established in human resource policies in the overall system of the national bureaucracy, opinions on sectoral differences were low. Variations in opinions within countries, on the other hand, would be linked to both variations in governance systems (e.g. centralised or decentralised political systems) as well as institutional factors (e.g. transparency in decision-making processes).

Bauhr & Charron [14] argue that perceptions of corruption are negatively associated with QoG and increase support for redistribution within the EU. The support is even more acute in contexts where the QoG is low, and there is poor public service delivery. Consequently, boosting support for cohesion policy is highly appreciated in contexts where the EU could be seen as a ’potential saviour’.

The authors finally noted that citizens perceive EU institutions as less corrupt than national institutions in contexts where the overall QoG is low, and in line with the "trade-off" hypothesis, citizens preferred centralised policies. Similarly, in a study conducted in Spain, Kuhn & Pardos-Prado [30] stated that corruption and the QoG in different geographical settings are key determinants of preferences for more or less decentralisation. They found that corruption at the national level decreases satisfaction with national policies, increasing preferences for decentralisation and secessionist self-determination [31] leading citizens in highly corrupt regions to prefer reducing the national domains of unitary states. The trade-off hypothesis has also been used in the literature as a consistent argument that explains support for European integration [32–34].

Suzuki and Demircioglu [35] pointed out that, in the public policy arena, government impartiality or neutrality is often seen as a central feature of QoG. The authors defined impartiality in official and administrative processes as the equal treatment of people regardless of differences in race, gender and other socio-demographic characteristics. In line with Grabham [36], it is necessary that rules and expectations about public services are transparent and equally applicable to all in the manner previously stipulated in laws and regulations.

Nevertheless, the authors found that strict standards of neutrality and impartiality could harm the most vulnerable citizens, but the lack of impartiality could have negative consequences such as corruption and trust reduction. They pointed out that "formal equality" treatment could be detrimental to "substantive equality/equity" without considering various forms of positive discrimination with social origins. Thus, citizens with a socially disadvantaged status might have less to gain from public services when the government emphasises such tight fairness [37].

However, it is expected that laws and regulations provide for exceptions that ensure that all citizens have access to administrative processes based on real and effective equality. Thus, it is clear that high impartiality would imply that public servants follow impartial or impersonal rules rather than biased or personal ones in handling individual administrative cases [38]. Consequently, decisions on individual cases are subject to written rules and regulations stipulated in policies or laws, rather than personal philias or phobias and relationships [15]. Values such as neutrality, equal treatment before the law and compliance with rules and law are essential principles of public servants in Max Weber’s classical bureaucracy [39].

### 2.1 Datasets to measure QoG

The World Governance Indicators dataset provides information on the quality of governance of a set of 200 countries provided by firms, citizens and a panel of experts. The data are compiled on six broad dimensions of governance: (1) Voice and Accountability; (2) Political Stability and Absence of Violence; (3) Government Effectiveness; (4) Regulatory Quality; (5) Rule of Law; and (6) Control of Corruption. The dataset is gathered from some survey institutes, think tanks, non-governmental organizations, international organizations, and private sector firms [40].

The Transparency International Corruption Perceptions Index (CPI) [41] ranks a group of countries and territories by their perceived levels of public sector corruption, according to experts and business people. For example, the 2020 CPI dataset highlights the impact of corruption on government responses to COVID-19 and compares the performance of the countries with respect to the investment in health care and the extent to which democratic norms and institutions have been weakened during the pandemic.

The European Quality of Government Index (EQI) dataset compiles information on the quality of government, collecting individuals’ opinions in NUTS2 regions in all the member states of the EU. Three aspects of QoG are included in the dataset: (1) impartiality, (2) corruption, and (3) quality. The 2021 edition includes several COVID-19 questions to analyse how the pandemic has affected the QoG [42].

## 3 Data

It was already commented that studies on impartiality have been possible for the recent development of new datasets, such as the one used in the study performed by the Quality of Government Institute in Gothenburg, Sweden. The institution provides three main types of datasets: systematic compilation, individual contributions and the European Quality of Government Index (EQI). All the QoG datasets can be accessed from Data Downloads section on the QoG Website: https://qog.pol.gu.se/data/datadownloads. The study uses the last EQI survey round of 2021 [42].

The survey was administered by a French market-research company via computer-assisted telephone interviews (CATI), complemented for the first time with online interviews. Sample representativeness is sought using the “next-birthday method” for the CATI subsample substituting the traditional quotas method. The “next-birthday method” consists of interviewing the person in the household older than 18 years old who will have the next birthday. The online subsample is based on the standard quota method. Charron et al. [42] contended that the quota method is stronger in terms of demographic representativeness but worse at finding a more ample range of opinions than the next-birthday method.

The interviews took place from October 2020 to February 2021, and 129991 respondents from the EU were finally interviewed. The sample was significantly expanded, and the dataset provides comprehensive information at the nomenclature of territorial units for statistics NUTS2 level for all the 238 EU regions. Due to Brexit, the 2021 sample did not include the regions from the United Kingdom. The survey asked questions about the quality, impartiality, and corruption of their public administration in order to get a general picture of how QoG is perceived by citizens.

The study will mainly use the questions related to impartiality regarding the public school system, public health care system and police forces. Thus, special emphasis is given to the following questions’ answers: (A1) Certain people are given special advantages in the public education system in my area; (A2) Certain people are given special advantages in the public health care system in my area; (A3) The police force gives special advantages to certain people in my area; (E1) All citizens are treated equally in the public education system in my area; (E2) All citizens are treated equally in the public health care system in my area; and (E3) All citizens are treated equally by the police force in my area.

The advantage questions are reverse recode and the answer format is based on a ten-point anchored Likert scale from 1 Strongly agree to 10 Strongly disagree. The equal questions are also reverse recode and the answer format is based on a four-point Likert scale as 1 disagree, 2 rather disagree, 3 rather agree and 4 agree. The reverse recode format is used to express the idea that higher figures are aligned with higher institutional impartiality. The answer format and question-wording open the opportunity to research two important issues that are frequently researched in survey design such as the number of points in the answer format [43,44] and whether the use of negative wording is adequate [13,45,46].

Likert scale is still the most popular answer format used in survey design. Rennis Likert introduced the scale for the first time in 1932 with the initial wording as strongly approve, approve, undecided, disapprove, and strongly disapprove [47]. The scale we name today as a Likert scale changed from the original word approve to agree, and many variations of the seminal scale exist today. Two of the existing variations are analysed in the study. Charron et al. [42] did not use a mid-point in the scales so it is forcing the respondents to make a favourable or unfavourable opinion about the impartiality of each institution. Thus, it is possible that some missing values could have their origin in that respondents do have a neutral opinion, and they cannot decide to make an opinion that is charged with a positive or negative impartiality feeling.

Table 1 shows the cross-tabulation of the responses for A1 and E1. There are interesting results that deserve our attention. First, it can be seen that both scales are not independent, and there exists a positive association that justifies the subjacent hypothesis that both questions measure the same concept of impartiality in public education. Second, 10,691 respondents agree strongly with the fact that certain people are given particular advantages in the public education system in my area, so, in principle, it would have been expected that respondents would have disagreed with the fact that all citizens are treated equally in the public education system in my area. However, there are only 4,092 respondents with such response behaviour. It is certainly true that the cognitive burden of the equality questions is higher than the parallel advantage question. It does not seem reasonable to observe such inconsistent answers, except for giving a positive connotation to the advantages given to some groups, but this would even create more problems in the ordinal nature of the impartiality concept measured by the advantage questions.

**Table 1:** Impartiality in public education.

*Advantages (A1)*	*Equality (E1)*					*Total*
	Disagree	Rather disagree	Rather agree	Agree	Missing	
***Strongly agree***	4092	2086	1686	2637	190	10691
***2***	934	1042	1046	858	85	3965
***3***	1962	2805	3111	2394	207	10479
***4***	1756	3290	3775	2411	242	11474
***5***	1210	2676	4149	2068	250	10353
***6***	2289	3662	7765	5353	582	19651
***7***	616	1088	3062	1544	97	6407
***8***	572	1105	4106	2698	112	8593
***9***	457	648	3234	3347	90	7776
***Strongly disagree***	1694	1399	4953	12522	350	20918
***Missing***	1345	2055	5987	4143	6154	19684
***Total***	16927	21856	42874	39975	8359	129991

χ^2^ = 45695.310 · df = 40 · Cramer’s V = 0.296 · p = 0.000.

Third, the other extreme of the advantage question, strongly disagree, shows fewer inconsistent results than the strongly agree case as there are 12,252 respondents who also agreed that all the citizens are equally treated. A similar analysis could have been done starting with the extreme responses given to the equal questions, as it seems inconsistent that if respondents agree or disagree with the fact that citizens are all treated equally, then they should not strongly disagree or strongly agree with the fact that certain people are given special advantages. And fourth, regarding the missing variables, 6,154 respondents seem to be consistent as they do not provide an answer for any of the questions. Meanwhile, 13,530 respondents did not answer the advantage question but they were capable of discerning whether the citizens were treated or not equally. On the other hand, 2,205 respondents did not answer the equally question but were capable of providing an answer to the advantage question. It seems that the number of points in the Likert scale is affecting the cognitive burden of the respondents.

## 4 Methodology

### 4.1 Fuzzy TOPSIS and fuzzy clustering

The section describes succinctly how two synthetic impartiality indicators are obtained for each of the perceptions, namely advantage and equal, using the three items, public education, health care service and police forces. The items were responded to on different scales and contained vague information. Thus, the study will apply a hybrid fuzzy TOPSIS that has been applied in different contexts, such as airlines satisfaction [48], hotels’ satisfaction [49], tourist destinations [50], national identity [51] and attitudes toward immigrants [52].

Leon and Martin [48] contended that hybrid fuzzy TOPSIS methods are becoming very popular in multi-criteria decision-making (MCDM) studies for several reasons: (1) they are flexible and easy to use in handling vague information; (2) they provide a more intuitive connotation than other MCDM methods; (3) they can be adapted to different objective function without making a strong assumption on the dimensionality of the problem.

Table 2 shows the scale conversion from the impartiality Likert format answers to triangular fuzzy numbers (TFNs). The transformation of the Likert scales provided by the interviewees, from 1 to 4 (impartiality-equality), and from 1 to 10 (impartiality-advantages) into TFN contained in the range of the discourse [0, 100] assumes that the nature of the information is imprecise. The essence of this conversion is that the 3-coordinate vectors (*a*_1_,*a*_2_,*a*_3_) that represent each consecutive answer have a non-empty intersection. For example, it can be seen that disagree and rather disagree in the impartiality equality scale intersect in the interval (0,30), while strongly agree and 2 in the impartiality advantages scale intersect in the interval (0,10).

**Table 2:** Likert scales conversión.

*Impartiality Advantages (A1-A3)*	*TFN*	*Impartiality Equality (E1-E3)*	*TFN*
***Strongly agree***	(0, 0, 10)	***Disagree***	(0, 0, 30)
***2***	(0, 10, 20)	***Rather disagree***	(0, 30, 60)
***3***	(10, 20, 30)	***Rather agree***	(40, 70, 100)
***4***	(20, 30, 40)	***Agree***	(70, 100, 100)
***5***	(28, 40, 53)		
***6***	(48, 60, 73)		
***7***	(60, 70, 80)		
***8***	(70, 80, 90)		
***9***	(80, 90, 100)		
***Strongly disagree***	(90, 100, 100)		

The membership function *μ*_*A*_(*x*) for the triangular fuzzy numbers (*a*_1_,*a*_2_,*a*_3_) of Table 2 is as follows:

$$\mu_{A}(x)=\{\begin{array}{l} \frac{x-a_{1}}{a_{2}-a_{1}},\;a_{1}\leq x\leq a_{2},\\ \frac{x-a_{3}}{a_{2}-a_{3}},\;a_{2}\leq x\leq a_{3},\\ 0,\;otherwise. \end{array}$$ (1)

The membership function represents the truth degree of each of the responses, and it can be seen that the maximum truth is given to the intermediate value of the coordinates of the TFN.

A synthetic impartiality indicator (IMP index) for each respondent can be calculated following the Technique for Order Preference by Similarity to Ideal Solution (TOPSIS). TOPSIS is one of the most employed multi-criteria decision-making techniques [53,54]. The method is computed as follows:

$$\begin{array}{l} A^{+}=\{(\max V_{ij}|j\in J),(\min V_{ij}|j\in J^{\prime }),i=1,2,\ldots ,m\}\\ A^{-}=\{(\min V_{ij}|j\in J),(\max V_{ij}|j\in J^{\prime }),i=1,2,\ldots ,m\} \end{array}$$ (2)

where *J* and *J´* divide the different indicators included in the impartiality scale using the perspective of advantages or equality respectively according to benefit or cost characteristics. In our case, for each of the perspectives employed, it can be seen that the three indicators included in each scale can be considered a benefit.

Once the ideal solutions are calculated according to Eq 2, the relative IMP index for each citizen can be calculated with the help of the distances obtained between each observation and the respective ideal solutions [53,54]. Thus, the impartiality index can be obtained according to:

$$\begin{array}{l} S_{i}^{+}=dist(V_{i},A^{+})=\sqrt{\sum_{j=1}^{n}{\left(V_{ij}-A_{j}^{+}\right)}^{2}}i=1,2,\ldots ,m\\ S_{i}^{-}=dist(V_{i},A^{-})=\sqrt{\sum_{j=1}^{n}{\left(V_{ij}-A_{j}^{-}\right)}^{2}}i=1,2,\ldots ,m\\ IMP_{i}=\frac{S_{i}^{-}}{S_{i}^{+}+S_{i}^{-}}i=1,2,\ldots ,m \end{array}$$ (3)

where 0≤*IMP*_*i*_≤1 is now the impartiality synthetic indicator for citizen *i*. Thus, a citizen finds that the three public services under analysis, public education, health care units and police forces, are more impartial whenever the relative index is closer to 1. TOPSIS is a relative concept which bases the optimality in the fact that the best alternatives should have the shortest distance from the positive-ideal solution and the longest distance from the negative-ideal solution. Thus, the impartiality indices for each citizen could be used to rank citizens’ impartiality perception from more or less impartiality according to the descending order of *IMP*.

The comparison and the analysis of impartiality using the negative wording of advantages and the positive wording of equality at the individual level will be based on Fuzzy Clustering [48]. Thus, the membership function can determine the degree of similarity that each citizen has with respect to a profile representing a representative group [52]. On certain occasions, D’Urso et al. [55] contended that it is better to obtain a three-cluster solution rather than an optimal solution that is based on some optimal loss function that points out to have more than three clusters. Sometimes, it is better to obtain a graphical representation based on those who perceive the public services as extremely impartial, extremely partial and intermediate impartial. More clusters will probably obscure this simple but effective representation.

Three representative citizens’ profiles are obtained according to the maximum, minimum and median observation found by *IMP* indices. The fuzzy segmentation analysis extends that of the Bagged Cluster algorithm introduced by Leisch [56], and can be mathematically expressed as follows:

$$\{\begin{array}{c} \min:\sum_{i=1}^{n}\sum_{c=1}^{C}u_{ic}^{m}d_{F}^{2}({\tilde{x}}_{i},{\tilde{p}}_{c})=\sum_{i=1}^{n}\sum_{c=1}^{C}u_{ic}^{m}[w_{2}^{2}{\left\|a_{2}^{i}-p_{2}^{c}\right\|}^{2}+\\ +w_{1}^{2}({\left\|a_{1}^{i}-p_{1}^{c}\right\|}^{2}+{\left\|a_{3}^{i}-p_{3}^{c}\right\|}^{2})]\\ s.t.\begin{array}{c} m>1,u_{ic}\geq 0,\sum_{c=1}^{C}u_{ic}=1,\\ w_{1}\geq w_{2}\geq 0,w_{1}+w_{2}=1 \end{array} \end{array}$$ (4)

where $d_{F}^{2}({\tilde{x}}_{i},{\tilde{p}}_{c})$ is the squared fuzzy distance between the *ith* citizen and the profile of a set of representative citizens ${\tilde{x}}_{i}\equiv (a_{1ik}a_{2ik}a_{3ik}):k=1\ldots K$ where the vector represents the TFN assigned to the information provided by the i-th citizen [57]. ${\tilde{p}}_{c}\equiv \{{\tilde{p}}_{ck}=(p_{1ck},p_{2ck},p_{3ck}):k=1\ldots K\}$ represents the TFN provided by the representative citizen of the cth cluster, while the expression ${\left\|a_{2}^{i}-p_{2}^{c}\right\|}^{2}$ is the squared Euclidian distances between the centers of the TFN vectors of the ith citizen and the representative citizen of the cth cluster. The squared Euclidian distances between the left and right extreme components of the TFN vectors of the ith citizen and the representative citizen of the cth cluster are represented by ${\left\|a_{1}^{i}-p_{1}^{c}\right\|}^{2}$ and ${\left\|a_{3}^{i}-p_{3}^{c}\right\|}^{2}$. In addition, *w*_1_ ≥ *w*_2_≥0 are suitable weights respectively for the centre, and extreme components for the fuzzy distance considered, and the weighted exponent that controls the fuzziness of the obtained partition *m* is larger than one. Thus, the membership degree of the *ith* citizen in the *cth* cluster is given by *u*_*ic*_ and it is obtained by the Lagrangian minimization problem. For more information on cluster validation and cluster profiles, readers can consult [55,58,59].

The fuzzy clustering method segments the whole set of citizens into a set of a metric that determines the resemblance degree of the citizen with respect to the three mentioned citizens’ profiles [60,61]. The coefficient m is known as the “fuzziness coefficient” and measures the fuzziness degree that directly increases with m, being minimum when m is equal to one. In the study, m is equal to 1.5 as in [52,55].

### 4.2 The eco-extended apostle model

Schaefer [62] extended the "apostle model" to facilitate the ecological understanding of invasive species, species at risk, and keystone species. Thus, the classical model quadrants introduced in the mid-1990s by the Harvard Business School (Jones and Sasser [63]) to analyse the relationship between loyalty and satisfaction as a way to improve the survival rate of the organizations was transformed into ecological terms. Thus, the four quadrant and their respective classical categories originally denominated as deserters, mercenaries, hostages and apostles, deserters, could be transformed in ecology transferring the following concepts: products as habitats and clients as species. More recently, the eco-apostle method has also been used by Indelicato and Martín [51] to analyse the four categories of citizens using civic and ethnic dimensions of national identity as the old clients’ pair loyalty-satisfaction. The latter study used also a fuzzy clustering technique to extend the four categories into sixteen more refined categories.

Similar to what Schaefer [62] did in ecology and Indelicato and Martín [51] did in social science studying national identity, the current study will transform the four classical categories into sixteen new categories that will be used to analyse how citizens are split into the bi-dimensional space according to impartiality under the two perspectives in which the perspective of the advantages will take the role of satisfaction and the equality perspective will take the role of loyalty. Thus, the model will distinguish between those who responded consistently to the impartiality construct independently of using positive or negative wording, from those who were not consistent manifesting that impartiality is low or high depending on the perspective used to analyse the concept.

**Fig 1:**
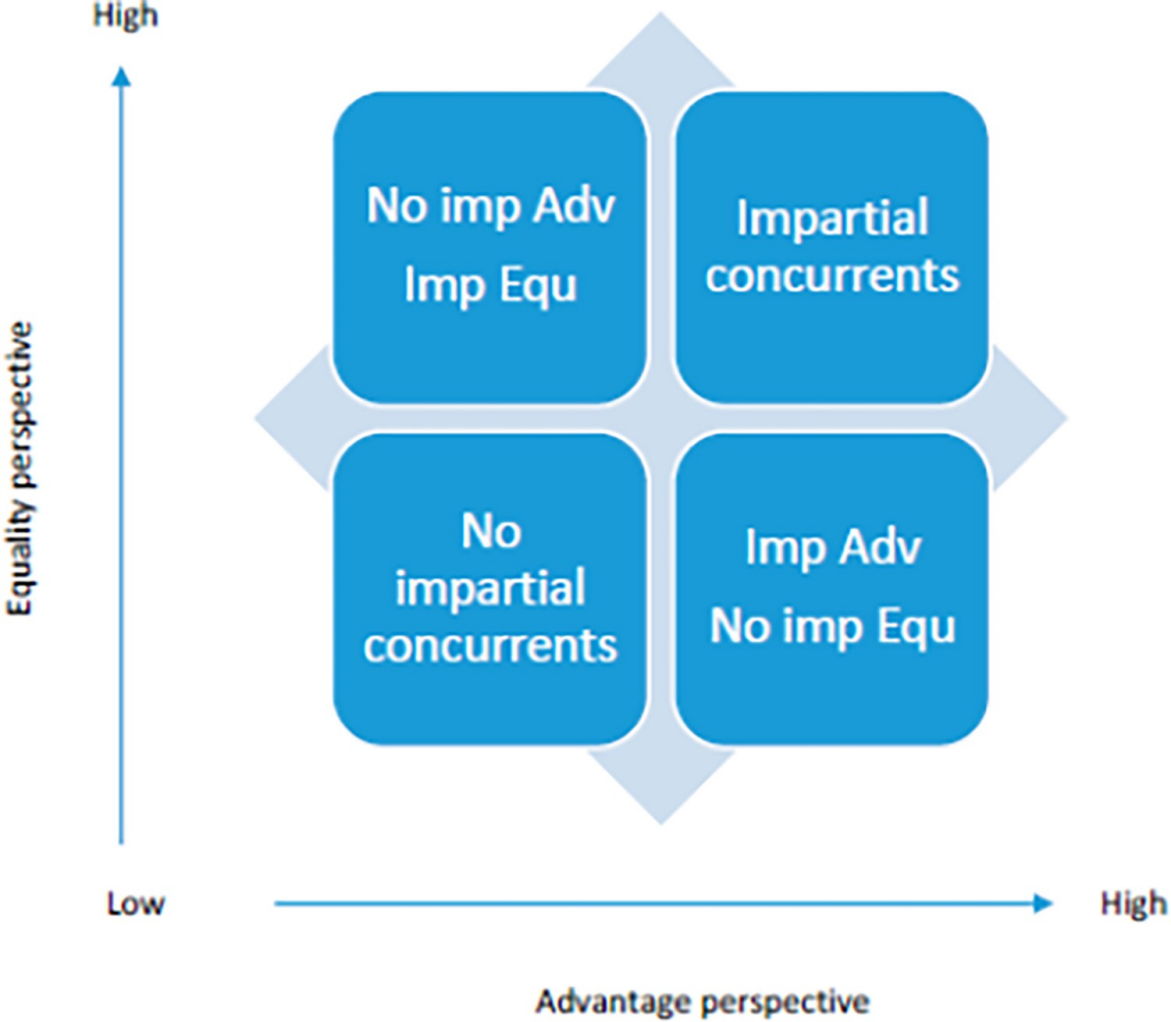
Apostle model applied to impartiality.

Fig 1 shows the classical four quadrants explained above through the IMP-TOPSIS indicator at an individual level. Thus, the south-west quadrant (deserters) can be seen as the citizens who concur that the provision of public services is not impartial. Similarly, the north-east quadrant (apostles) is characterized by those citizens who perceived that the provision of public services was made with impartiality. The south-east and north-west quadrants are the most controversial from the point of view of survey design as they contain citizens who expressed inconsistent opinions about impartiality depending on whether the question is made with a perspective of equality (positive wording) or advantages (negative wording).

A limitation of this method is that there are some observations who are very close to the average IMP TOPSIS values, and the separation of the quadrants for these observations is sometimes unclear or inappropriate. Thus, near the average values, it would be more difficult to determine whether the citizens are providing consistent or inconsistent answers, and, for this reason, the new proposal based on the ternary graphs obtained after the fuzzy clustering analysis following Indelicato and Martin [51] will be used in the study. The extended eco-apostle model refines the blurred observations obtained with the classical model.

The new model extends the four classical categories into sixteen different classes that are determined by the membership functions of the fuzzy clustering method. Among these, the four corner categories determined by whether the citizens are more similar to those who perceive the public provision as the most or least impartial under the two perspectives will be further analysed with binary probit models. Special emphasis will be put on the controversial quadrants because they contain citizens who provided the largest inconsistent answers.

As said, the extended method is based on the membership functions obtained for the analysis of impartiality under the advantages and equality perspectives. Thus, let us assume that *a* = (*a*_1_,*a*_2_,*a*_3_) and *e* = (*e*_1_,*e*_2_,*e*_3_) are the impartiality-advantages and impartiality-equality membership functions of a particular citizen. Following Indelicato and Martín [51], we define the following function for each membership function vector *m*:

$$f(m)=\{\begin{array}{c} 1\;if\;m_{2}>0.5\\ 3\;if\;m_{3}>0.5\\ 4\;if\;m_{1}>0.5\\ 2\;otherwise \end{array}\}$$ (5)

It can be seen that, without loss of generality, the function is transforming the ternary graph distribution into four equilateral triangles in which the upper triangle those who are more similar to the citizens who experienced the public provision as the least impartial takes the value of one. Similarly, the left-lower triangle which is characterized for those who are more similar to the citizen who sees the public provision as the most impartial gets the value of four. Finally, the right lower triangle and the middle triangle get the values of three and two, respectively. The lower triangle characterizes the intermediate position regarding the impartiality concept. Thus, the classical apostle model is extended to a model with 16 different categories in which the pure categories located in the corner quadrants are characterized by the following pairs ((1,1), (4,1), (1,4) and (4,4)). These four pure categories will be further analysed with the help of binary probit models. As previously said, the analysis of the pure controversial quadrants characterized by (4,1) and (1,4) will be of special relevance for the study.

Four binary ordered probit models will be estimated to analyse whether a respondent belongs to any of the four corner quadrants using the following twelve explanatory variables: country, gender, economic activity, nativity in the country, size of the city, public school contact, health care system recently used, police or security forces contact, age, level of education, main activity, and economic situation judgement.

After the ordinal probit models were estimated, marginal effects were then calculated to analyse how differences in the explanatory variables affected the probability of being citizens who perceive the service provision as partial in both perspectives, partial-equality and impartial-advantages, impartial-equality and partial-advantages, and impartial in both perspectives. Dummy coding is the most used method to normalize the categorical variables, but for reasons of interpretability, the study uses the effects coding proposed by Daly et al. [64] in which the marginal effects can be interpreted with respect to the sample average, and for that, the comparison between different explanatory variables are more valuable.

## 5 Results

We do not provide any descriptive statistics as these can be consulted in Charron et al. [65]. 129,991 respondents were selected to complete the QoG survey for the EU27 making that the sample was significantly expanded in comparison with previous years, and 238 NUTS2 regions were included in the sample. There were only five countries, Luxembourg, Estonia, Latvia, Malta and Cyprus, for which there was only one NUTS2 region. For the first time, after the Brexit, the sample did not include the United Kingdom in the dataset.

Table 3 shows the TFNs and defuzzified crisp values of the total sample. It can be seen that all the intervals of the discourse of the TFNs overlap. However, analysing the defuzzified crisp values, results show that impartiality is more highly valued under the equality perspective than under the advantages perspective. Thus, it can be inferred that the negative wording is affecting the citizens’ impartiality evaluation. Another interesting issue to highlight is that under the advantages perspective, the impartiality ranking shows that police forces are seen as the most impartial public service, followed by the education and the health system. Meanwhile, a different pattern is observed when the impartiality is analysed with the equality perspective. In this case, the public education is seen as the most impartial, followed by the police forces and the health system. The range of the TFNs for the equality perspective is also affected by the conversion of the 4-point Likert scale. It is also interesting to remark that at the overall EU27, there is only one impartiality indicator which seems to fail, the health system under the advantages perspective (49.29). In the rest of the cases, impartiality figures are higher than 50.

**Table 3:** TFNs and crisp values for the total sample.

Indicator	TFN	Crisp value
**Adv-Education**	(45.72, 55.43, 64.21)	55.20
**Adv-Health**	(39.98, 49.36, 58.47)	49.29
**Adv-Police forces**	(47.00, 56.55, 65.00)	56.28
**Equ-Education**	(46.02, 66.52, 84.13)	65.80
**Equ-Health**	(42.98, 62.42, 81.78)	62.40
**Equ-Police forces**	(43.45, 63.01, 82.11)	62.90

Table 4 shows the profiles of the 3-solution cluster according to selecting the maximum, minimum and median of the index *IMP* that was obtained for each of the perspectives, namely advantages and equality (Eq 3). Analysing first the impartiality from the advantages perspective, it can be seen that the profile of the citizens who see the public service provision as the most impartial answered each of the questions as strongly disagree showing that there are not citizens who are given special advantages. On the other hand, the profile for those who see the public service provision as the least impartial answered the questions as strongly agree to show that certain people are given special advantages. And finally, the moderate profile is characterized by showing more disagreement with education and police forces in comparison with the health care services. Nevertheless, the moderate impartial citizen profile is closer to the most impartial citizen than the least impartial citizen.

**Table 4:** Fuzzy cluster profiles.

Advantages	Most impartiality	Least impartiality	Interm. impartiality	Equality	Most impartiality	Least impartiality	Interm. impartiality
**Education**	10	1	6	**Education**	4	1	3
**Health**	10	1	5	**Health**	4	1	2
**Police forces**	10	1	6	**Police forces**	4	1	2

The analysis of the impartiality under the equality prism is very similar. The most and the least impartial citizen profiles are also characterized by the extreme positions of the citizens regarding the three services under analysis, but now they agree or disagree with the fact that all citizens are treated equally. Meanwhile, the intermediate profile is now characterized by experiencing more impartiality in education than in health care units and the police forces. It is interesting to note that now the intermediate profile is closer to the least impartial profile than the most impartial profile. Thus, it can be conclude that the profiles are affected by the wording and the number of points in the Likert scale.

**Fig 2:**
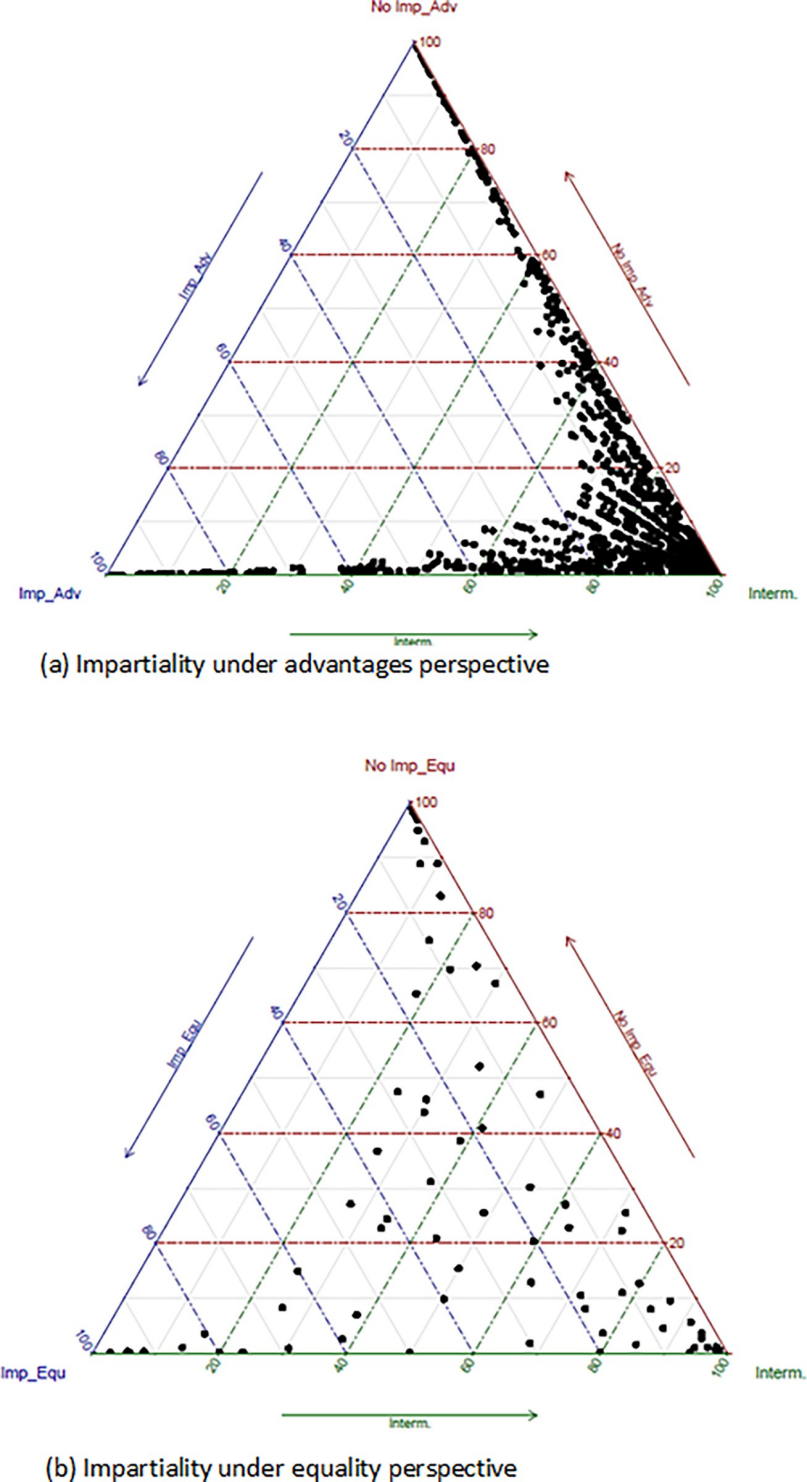
Fuzzy clustering ternary graphs.

Fig 2 shows the ternary graphs of the fuzzy clustering method for both impartiality constructs. The analysis of the advantages perspective shows that a majority of citizens are closer to the most impartiality profile with an aggregate membership value of 38.7 per cent. Meanwhile, 25.4 percent and 36.0 percent are similar to the least and moderate impartiality profiles. On the other hand, the graph that shows the citizens distribution according to the impartiality under the perspective of equality presents a different pattern than the case previously commented. It is interesting to see that now the majority group is represented by the intermediate impartiality cluster with an aggregate figure of 57.3 percent. Meanwhile, the most and least impartiality clusters are represented by 23.6 and 19.1 percent respectively. It seems that citizens are more similar when the number of points on the Likert scale is lower. It can be concluded that the questions’ wording and the number of points in the Likert scale is affecting the impartiality measurement.

**Fig 3:**
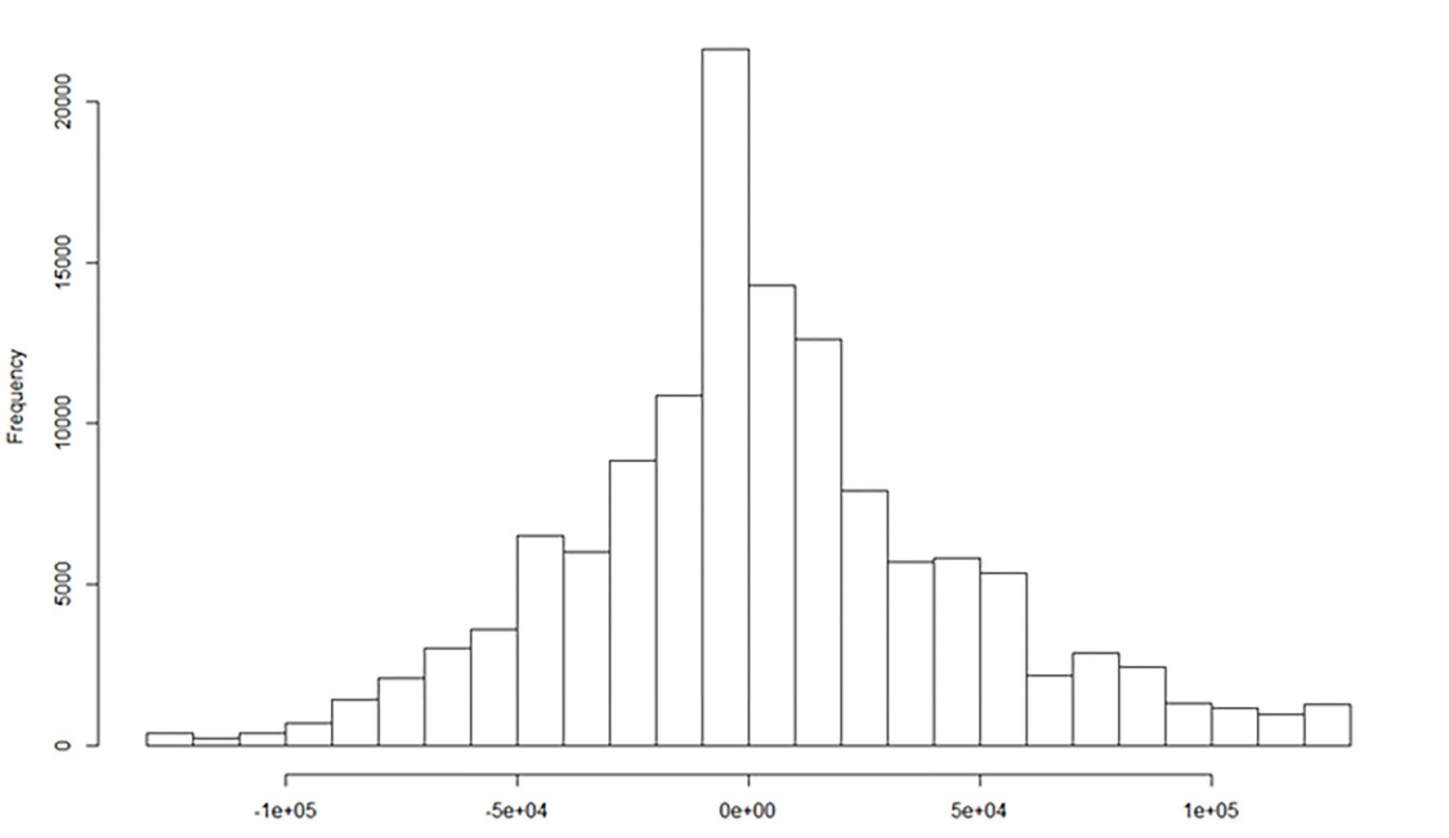
Histogram of the ranking difference between *TOPSIS IMP ADV* and *TOPSIS IMP EQU*.

Fig 3 shows the histogram of the differences obtained between the rankings of the *TOPSIS IMP* indicators using both perspectives: advantages and equality. The skew coefficient is 0.25, so we conclude that there is a positive trend to measure the impartiality more positively when the scale is based on the perspective of advantages, that is, citizens tend to evaluate impartiality more positively when they evaluate it answering whether certain people are given special advantages. The extreme differences are observed for individuals who answered both scales in an opposite way (10’s for all the attributes in the advantage scale and 1’s in the equality scale; and, vice versa, 1’s for all the attributes in the advantage scale and 4’s in the equality scale).

There are also interesting differences for some other groups of answers such as: (1) 10’s for all the attributes in the advantage scale and 2’s for the attributes in the equality scale; (2) some values lower than 10 in the advantage scale and 1’s for all the attributes in the equality scale; (3) other intermediate values that measure the extent to which the distribution has long-drawn-out tails; and (4) consistent rankings for a set with more citizens that are observed around the zero value.

The classic apostle model applied to the axes impartiality-equality and impartiality-advantages taking the roles of loyalty and satisfaction, respectively, and using the synthetic TOPSIS impartiality indicators forms the classical four quadrants. The four quadrants are denominated as follows: (1) deserters are now denominated as concurrent citizens experiencing that public services are offered with certain partiality; (2) mercenaries are now seen as those citizens who do not perceive consistently impartiality being more benevolent when they evaluate it with the advantages perspective; (3) hostages are the reciprocal case of the second group, that is, inconsistent citizens who perceive that the public services are offered with impartiality when they evaluate it with the equality perspective; and (4) the apostles are seen as the concurrent citizens who perceive that the public services are offered with impartiality. The results show that concurrent citizens account for the 66.6 per cent of the sample, 36.5 per cent perceive impartiality and 30.1 perceive partiality. 16.6 per cent of the sample form the south-east quadrant (impartiality using advantages and partiality using equality). And, 16.8 per cent can be considered as inconsistent opinions of citizens who perceive impartiality using equality and partiality if the perspective of the advantages is taken.

The application of the extended eco apostle model using a cut value of 0.5 provides the following different results. Quadrant south-west is now represented by 10.3 per cent of the sample, and 11,560 respondents are pure concurrent citizens who perceive that the public services are offered with partiality. The south-east quadrant is represented by 21.4 per cent of the sample, and there are only 2,337 citizens that can be considered inconsistent type I (impartiality using advantages and partiality using equality). The north-west quadrant is now represented by 8.2 per cent of the sample with 4,787 citizens who can be considered inconsistent type II (partiality using advantages and impartiality using equality). And finally, the north-east quadrant is the most represented of the total sample with a total of 61.2 per cent for which 17,102 citizens are considered as consistent opinions of those who perceive that the public service provision is offered with impartiality.

Tables A1-A4 in the annex show the marginal effects of the four ordered probit models estimated for each of the pure categories of interest. It is interesting to remark that all the twelve explanatory variables show significant marginal effects for some of the pure categories under analysis. For the ease of exposition, the study highlights the five main drivers and barriers for each of the estimated models.

Thus, it can be seen that for the south-west corner category (Table A1), the five main drivers for being in this category are determined by country (Romania, Poland, Bulgaria and Croatia) and by judging the personal economic situation as very bad. In all the cases, the marginal effects are higher than 6 per cent, and that means that when citizens are in one of these groups they have more than 6 per cent of probability of being in this category than the average sample citizen. On the other hand, the five main barriers are only determined by the country (Estonia, Finland, Netherlands, Czech Republic and Luxembourg). In this case, the marginal effects are always lower than -4 per cent. Besides the five main drivers and barriers, other variables such as occupation and age affect also the probability of belonging to this category. It is interesting that military personnel, police officers and teachers perceive less partiality than the average citizen.

The results for the south-east corner category, pure mercenaries in the classical apostle model parlance, show that the five main drivers are characterized by the country (Poland, Slovakia, France and Cyprus), and for those citizens who judge their personal economy as very bad. In all the cases, the marginal effects of being in this category are higher than 7 per cent. As discussed above, these results could be inconsistent answers to the survey as citizens are perceiving the impartiality of the public service provision very differently under the two perspectives used in the analysis, namely equality and advantages. For this reason, further analysis will be necessary in this case. The analysis of the main barriers provides that the country is still the most prominent feature with Finland, Estonia, Czech Republic and Lithuania as representative examples, and those citizens who consider that their personal economy is very good complete the set of the five main barriers. In all the cases, the groups have less probability of belonging to this category than the average citizen with negative marginal effects lower than -4.8 per cent.

The north-west corner category (pure hostages) completes the analysis of the counterpart class of inconsistent responses. In this case, citizens perceive partiality when they use the perspective of the advantages, and they change their perception towards impartiality using the equality perspective. In this category, it can be seen that the main drivers are again determined by the country (Bulgaria, Croatia, Portugal and Lithuania) and by the citizens who judge the personal economy as very good. In all the cases, the marginal effects are positive and higher or equal to 3.5 per cent. On the other hand, the main barriers are determined by the country (Luxembourg, the Netherlands, Finland, Cyprus and Sweden). In all the cases, the marginal effects are negative and lower than or equal to -2.4 per cent.

The north-east corner category corresponds to those citizens who concur that the service public provision is perceived as impartial independently of what perspective is used. The results now show that the main drivers to be in this category are characterized by the country (the Netherlands, Czech Republic, Estonia and Finland), and for those who are employed in law enforcement, police or fire-fighter. In all the cases, the marginal effects are positive and higher than or equal to 11.2 per cent. On the other hand, the main barriers for being in the category are characterized by countries (Croatia, Poland, Bulgaria and Romania), and for those citizens who consider that their personal economy is very bad. In all the cases, the marginal effects are negative and lower than or equal to -15 per cent.

A parsimonious analysis of the controversial quadrants by each explanatory variable used in the study regarding the significant positive effects shows that there are only eight categories for which the results could be biased as the perspective for which the impartiality is analysed plays a determinant role and some inconsistencies are observed. There are three country categories (Romania, Belgium and Portugal), two age categories (18–29 and 30–49), and the rest of the categories are represented by females, citizens who have been born in a foreign country, and citizens who have recent contact with the police. Other categories, such as France, Slovakia and citizens who live in cities larger than one million people, are only characterized for being more significantly included in the south-west corner quadrant. It is out of the scope of the current study, to analyse whether the impartiality latent variable of some of the groups under analysis would be affected if these citizens are discarded from the analysis.

## 6 Discussion and conclusions

Impartiality is one of the features that jointly with the quality of public service provision do not correspond to any prewritten law that measures the outputs in objective terms, and for that reason, scholars usually rely on surveys opinions (Charron & Lapuente, [65]). Van de Walle and Migchelbrink [4] contended that the survey opinions are subjective, but fully objective indicators that measure QoG at the regional or national level are inexistent. Impartiality is seen as a necessary condition for a good QoG, but the lack of objective data regarding whether citizens consider themselves impartially treated independently of their social, economic, political, ethnic or religious position, makes its study a difficult task not exempt of criticism [66,67].

In the current study, another important criticism could be rooted in the data period mainly based on the COVID period. Thus, it is quite possible that some individuals may not be entirely rational while judging the quality of the government due to the Covid-related measures taken by their respective governments, such as, for example, lockdown and travel restrictions, among others. In this case, it would also be interesting to analyse to what extent the results could also be biased and sample-specific, and this could be an interesting line for future research using more recent datasets that are less affected by the commented pandemic period.

Let us imagine that we could have objective data of the percentage of population that have been discriminated in some of the public policy areas under study, namely education, health care and law enforcement. Let us assume that we know that 5 per cent of the population get some advantages in one public service, it would be interesting to analyse how citizens answer to the questions knowing this objective information. This information will provide a common heuristic rule of thumb known as anchoring, and the answers could also be affected by the internal adjustments made by the citizens regarding the level of agreement with the impartiality construct. Epley and Gilovich [68] showed that the adjustments could vary when the anchors are self-generated or induced. Thus, it would be very interesting to have self-generated anchors for a group of citizens answering the questionnaire with the advantages and equality perspectives to see to what degree, the anchors would limit the inconsistent answers.

The self-generated anchors will also provide adequate information to analyse the observed differences between the answers given to the positive (equality) and negative (advantages) questions wording. It is not risky to anticipate that citizens will answer differently to the anchors “10 of one hundred get some advantages” than to “ninety of one hundred are treated equally”. This is known as the framing effect [69], which is one of the most striking cognitive biases for which citizens react differently to a particular choice depending on whether the question wording invokes losses or gains in respondents’ minds. The framing effects are exacerbated because human nature restraints reframing that search for consistency in answering as most of us would not know what to do in case of contradiction [70].

The results of the eco-extended apostle model are consistent with the framing effects commented above as there are more inconsistent type II citizens (4,787) than type I (2,337). Inconsistent type II citizens are characterized by a different perception of impartiality when they analyse the public service provision with the negative wording using the perspective of the advantages. Meanwhile, the number of inconsistent type I citizens is inferior, that is, those who perceive impartiality using the advantages perspective and partiality using the equality perspective are less in number. These results are not previously found in the literature because individual data on impartiality using both perspectives have not been analysed up to now.

The analysis of the south-west pure corner quadrant (consistent citizens who experienced the public service provision as very partial) concludes that there is a solid duality between some Eastern countries such as Romania, Poland, Bulgaria and Croatia and some other Nordic and Western countries like Estonia, Finland, Netherlands, Czech Republic and Luxembourg. The first group is characterized by presenting a higher probability of belonging to this category, and the second group on the contrary presents a lower probability than the average sample citizen. It is also interesting to highlight that the citizens who considered as their economic situation as very bad had also a higher probability of belonging to the group of perceiving the public service provision as highly partial. This result is similar to that found by Van de Walle and Migchelbrink [4], when respondents who experience a more economic strain situation have a lower trust in public administration finding that the effect increased aligned with the economic strain situation.

Regarding the south-east pure corner group represented by citizens who provided inconsistent answers about impartiality, results show that citizens residing in Poland, Slovakia, France and Cyprus, jointly with those who judged their personal economy as very bad are overrepresented in the set. On the other hand, citizens residing in Finland, Estonia, Czech Republic and Lithuania as well as those citizens who considered that their personal economy was very good are underrepresented. It is out of the scope of the current study, but it seems obvious that some observations should be discarded as they present some noisy answers, so a word of caution should be given for some of the previous analysis [1,71,72].

Some more inconsistent answers are also observed in the north-west pure corner quadrant characterized for those who experienced the public service provision as partial only under perspective of the advantages that are obtained by some citizens. The group is overrepresented by those residing in Bulgaria, Croatia, Portugal and Lithuania and by the citizens who do not observe any economic strain. On the other hand, the group is underrepresented in the subsamples obtained in Luxembourg, the Netherlands, Finland, Cyprus and Sweden. This contradictory behaviour opens a new line for future research in order to discern whether some answers are invalid for a cognitive burden or because, for some citizens, advantages and equality are not exact opposite extremes when they evaluate impartiality. The analysis of cutoffs regarding the percentage of people who get some advantages, and the study of some control treatments that explain whether the advantages are based on positive discrimination or the bad behaviour of the institutions will also be interesting lines for future research.

The north-east pure corner quadrant corresponds to those citizens who provided consistent and extreme answers about the impartiality experienced by the public service provision. It is interesting to remark that for the first time, one of the main drivers is obtained for those employed in law enforcement, police or fire-fighter. The other four categories reside in the Netherlands, Czech Republic, Estonia and Finland. Finland and the Netherlands were found to be countries in which there are no significant regional differences in trust in public administration [4]. It is important to highlight that the authors found that the impartiality of public services is positively related to citizens’ trust in public administration and used regional data instead of national data for the multilevel analysis. Our study analyses the individual data trying to obtain the main drivers and drawbacks for the pure categories analysed. There is a less number of citizens in the category for those residing in Croatia, Poland, Bulgaria and Romania, and for those experiencing economic strain. The economic strain effects were already obtained for those who experienced that the public service provision was processed with partiality. It is usually assumed that economic strain situation makes the citizens more dependable on social welfare programs, and it becomes apparent that this issue can also affect on how citizens evaluate public service provision impartiality [73,74].

A final analysis to see whether some explanatory factor could explain a greater number of inconsistent impartiality answers showed that there were eight significant factors: three country categories (Romania, Belgium and Portugal), two age categories (18–29 and 30–49), as well as females, citizens who have been born in a foreign country, and citizens who have recent contact with the police. Other factors such as France, Slovakia and citizens who live in cities larger than one million people could explain only inconsistent answers included in the south-east corner quadrant. Future research could explore whether the elimination of some inconsistent answers using some threshold that can be determined in Fig 3 could affect the impartiality index obtained in some citizens’ groups. It is out of the scope of the current study to analyse to what extent discarding these a-priori inconsistent answers will change some basic results of the study of impartiality. Nevertheless, our study showed empirical evidence that impartiality is highly dependent on the wording of the questions or the format of the answers for some respondents. Thus, it is important for policymakers to exercise caution when comparing observed differences based on demographic covariates such as country, gender, native status, town size, occupation, and perception of the economic situation.

Response inconsistency can be the product of a natural cognitive burden, and not a meditated response distortion (faking) in which some respondents tend to respond in a way that creates a positive image of themselves under certain circumstances. Some authors have provided enough empirical evidence about the unexpected and negative consequences of inconsistent answers, such as biased intra-group scores, incorrect scales validity, and distorted rankings [75,76]. There are some attempts to mitigate these problems, especially in the literature on applicants’ job selection, but to our knowledge, these attempts are inexistent in the current case, but in order to increase transparency, additional questions about the percentage of the population who get special advantages preventing the thoughts of positive discrimination could ameliorate the number of inconsistent answers. The effectiveness of this strategy could deserve more research attention.
